# TO CHOOSE OR NOT TO CHOOSE: CAN AFFECTIVE OR COGNITIVE MECHANISMS EXPLAIN AGE DIFFERENCES IN DECISION AVOIDANCE?

**DOI:** 10.1093/geroni/igad104.0094

**Published:** 2023-12-21

**Authors:** Julia Nolte, Corinna Löckenhoff

**Affiliations:** Tilburg University, Tilburg, Noord-Brabant, Netherlands; Cornell University, Ithaca, New York, United States

## Abstract

Older adults are more likely to avoid decisions, which puts them at risk of experiencing negative consumer or health outcomes. Because the underlying reasons remain unclear, this pre-registered online study assessed the roles of (1) affect (especially regret), (2) subjective difficulty, and (3) cognitive ability in explaining age-related differences in decision avoidance. In addition, half of all participants were randomized to a writing intervention aimed at reducing avoidance tendencies. An adult lifespan sample (N = 432, Mage = 50.94, SDage = 19.53 years, 50% women, 62% Non-Hispanic White) chose between making or avoiding four consumer and health-based decisions. Affect, including regret, was assessed before (pre-decisional), during (peri-decisional), and after making decisions (post-decisional). Subjective difficulty was measured during the decision process. Finally, participants completed assessments of demographic background, socio-emotional experience, health, personality, and cognitive ability. We found that older adults were more likely to avoid decisions (p<.001), but age was unrelated to peri-decisional affect and subjective difficulty. Peri-decisional and post-decisional affect were not consistently related to avoidance, but participants who reported more difficulty were more likely to avoid decisions (p=.002). Covariates and cognitive measures could not explain age-related differences, but lower growth-goal-orientation and stronger maximization tendencies predicted decision avoidance after accounting for age (ps<.05). The intervention was successful at improving peri-decisional cognitive load and peri-decisional affect (ps<.05) but did not reduce decision avoidance. In sum, none of the assessed mechanisms could explain age-related increases in decision avoidance. Across age groups, perceived difficulty but not actual cognitive ability predicted avoidance tendencies.

